# Milk Injected into the Veins

**Published:** 1844-02

**Authors:** 


*Milk injected into the Veins.*—M. Donnè, in the course of the past summer, stated to the French Academy of Sciences, that, having been struck with the analogy between the constitution of milk and that of the blood, he had repeated the experiment of injecting the former fluid into the veins of various animals; and his experience, contrary to that of former observers, went to provê that those animals (except, from some unascertained cause, the horse), not only supported the operation without injury, but that the globules of milk served the purpose of those of chyle, and became eventually transformed into blood globules.—*Lancet*, from *Gazette des Hôpitaux.*

